# The Oldest Caseid Synapsid from the Late Pennsylvanian of Kansas, and the Evolution of Herbivory in Terrestrial Vertebrates

**DOI:** 10.1371/journal.pone.0094518

**Published:** 2014-04-16

**Authors:** Robert R. Reisz, Jörg Fröbisch

**Affiliations:** 1 Department of Biology, University of Toronto Mississauga, Mississauga, Ontario, Canada; 2 Museum für Naturkunde, Leibniz-Institut für Evolutions- und Biodiversitätsforschung, Berlin, Germany; 3 Institut für Biologie, Humboldt-Universität zu Berlin, Berlin, Germany; Raymond M. Alf Museum of Paleontology, United States of America

## Abstract

The origin and early evolution of amniotes (fully terrestrial vertebrates) led to major changes in the structure and hierarchy of terrestrial ecosystems. The first appearance of herbivores played a pivotal role in this transformation. After an early bifurcation into Reptilia and Synapsida (including mammals) 315 Ma, synapsids dominated Paleozoic terrestrial vertebrate communities, with the herbivorous caseids representing the largest vertebrates on land. *Eocasea martini* gen. et sp. nov., a small carnivorous caseid from the Late Carboniferous, extends significantly the fossil record of Caseidae, and permits the first clade-based study of the origin and initial evolution of herbivory in terrestrial tetrapods. Our results demonstrate for the first time that large caseid herbivores evolved from small, non-herbivorous caseids. This pattern is mirrored by three other clades, documenting multiple, independent, but temporally staggered origins of herbivory and increase in body size among early terrestrial tetrapods, leading to patterns consistent with modern terrestrial ecosystem.

## Introduction

The origin and early evolution of herbivory represents a major evolutionary event in terrestrial vertebrate history because its acquisition allowed tetrapods to become primary consumers, and access directly the vast, largely untapped resources provided by the primary producers on land, the cellulose rich leaves and stems of terrestrial plants. Herbivory in its broadest term refers to a form of consumption in which organisms eat principally autotrophs. Among vertebrates this feeding strategy can be subdivided into many categories, including folivory, frugivory, granivory, but among early terrestrial vertebrates, it is feeding on leaves, stems, roots, and rhizomes that are most relevant because it has the potential to be reflected in the skeletal anatomy [1], [2]. Thus, here we use the term herbivory in a restricted sense, as a form of feeding strategy in which the majority of nourishment is derived through the breakdown of cellulose in the digestive system with microbial endosymbionts [3]. This type of utilization of cellulose rich tissues from primary producers is most frequently reflected in the fossil record in the form of dental modifications for cropping and oral processing, and skeletal autapomorphies in the form of a wide, barrel-shaped rib cage for accommodating an enlarged gut. These dental and skeletal features are therefore amenable to critical evaluation in fossils [4].

Even though the earliest tetrapods are Late Devonian (late Frasnian, 375 Ma) in age, they effectively ventured onto land only during the Early Carboniferous (Visean, 345-328 Ma) [5]. Evidence of herbivory, as defined above, first appears in the fossil record more then 30 million years later, near the Permo-Carboniferous boundary [2]. Upper Pennsylvanian (Late Carboniferous) and Lower Permian continental sediments document a crucial stage in the initial diversification of terrestrial vertebrates because the fossils recovered from localities throughout the paleoequatorial belt of Pangaea in the southern part of Laurasia include not only some of the oldest known amniotes, but also the first herbivores and first large top predators [6]–[8]. The initial stage of the great amniote radiation is characterized by a dichotomy into synapsids (including extant mammals) and reptiles (including all extant reptiles and birds), two major clades that extend over 315 million years of evolutionary history. Interestingly, among Paleozoic amniotes, it is synapsids that evolved rapidly, and diversified greatly to dominate the terrestrial ecosystems of their time, while reptiles were initially less prominent and diverse [6]. Thus, the early evolutionary history of synapsids is of particular interest and critical to our understanding of the initial stages of amniote evolution and the origin of herbivory.

It is generally recognized that two clades of early synapsids include herbivores, the caseids and edaphosaurs [1], [2]. These two clades are each part of more inclusive synapsid lineages that comprise basal faunivorous and predatory members, suggesting that herbivory evolved more than once during the initial stages of synapsid evolution [2]. Herbivorous members of these two clades have different feeding strategies, with little oral processing being employed by the caseids having non-occluding, leaf-shaped dentition similar to those seen in the extant squamate *Iguana* [9], whereas extensive oral processing was present in edaphosaurs through the use of massive crushing dentition on the palate and mandibles [10]. While the fossil record of edaphosaurs and their evolutionary history is reasonably well known [10]–[12], the early history of caseids is shrouded in mystery [9].

Caseids belong to the most basal clade of Synapsida, the Caseasauria, which also includes the small carnivorous eothyridids (*Eothyris* and *Oedaleops*). Although evolutionarily significant because of their large size, caseid body fossils have been largely restricted to the later part of the Early Permian of North America and the Middle Permian of Western Europe and Northern Russia (276-264 Ma), while most of the other clades of early synapsids first appear within the Pennsylvanian (more than 300 Ma). Caseids are therefore some of the youngest pelycosaur-grade synapsids, representing only one of two clades (the other being Varanopidae) that survived to co-exist with the more derived therapsid synapsids [9], [13]–[15]. It appears that caseids may have even survived into the Late Permian in Europe, as indicated by the discovery of a very large caseid in the Lodève Basin [16]. These beds have been tentatively assigned to an early Lopingian age [17]. An erroneous recent claim of the presence of caseids in the latest Pennsylvanian of North America [18] is based on a single tooth, which does not conform to the well-known dental patterns seen in any known caseid. The fossil record of caseids has recently been extended by the discovery of two early Permian members of the clade [19], [20], reducing somewhat the ghost lineage of this clade.

**Figure 1:**
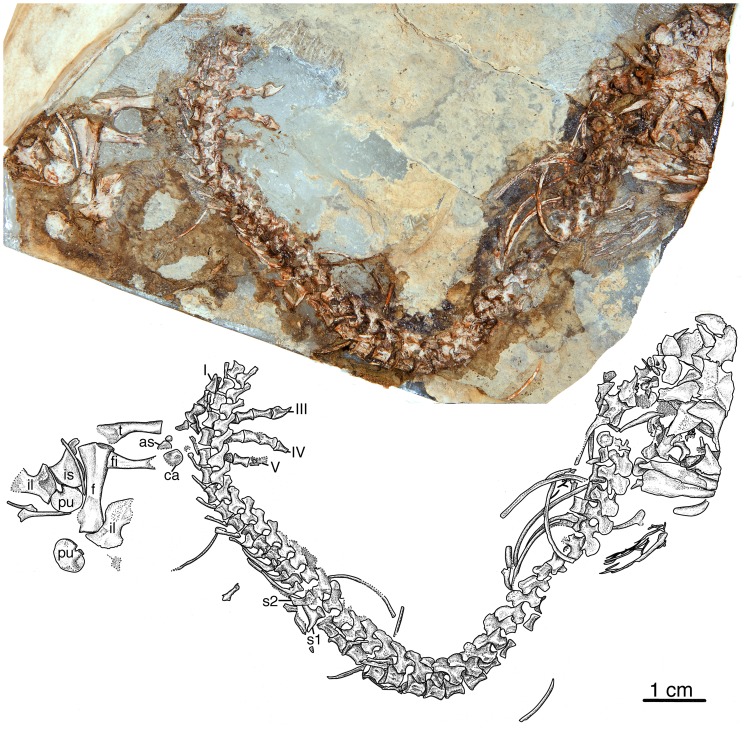
*Eocasea martini* gen. et sp. nov. Photograph and interpretive drawing of holotype KUVP 9616b, from the late Pennsylvanian of Kansas, USA. Preserved parts of the skeleton include the posterior 1/3^rd^ of the skull and mandible, the vertebral column with ribs, pelvic girdle and right hind limb. Abbreviations: as, astragalus; ca, calcaneum; f, femur; fi, fibula; il, ilium; is, ischium; pu, pubis; s1, first sacral rib; s2, second sacral rib; t, tibia; roman numerals indicate the digit number in the pes.

**Figure 2:**
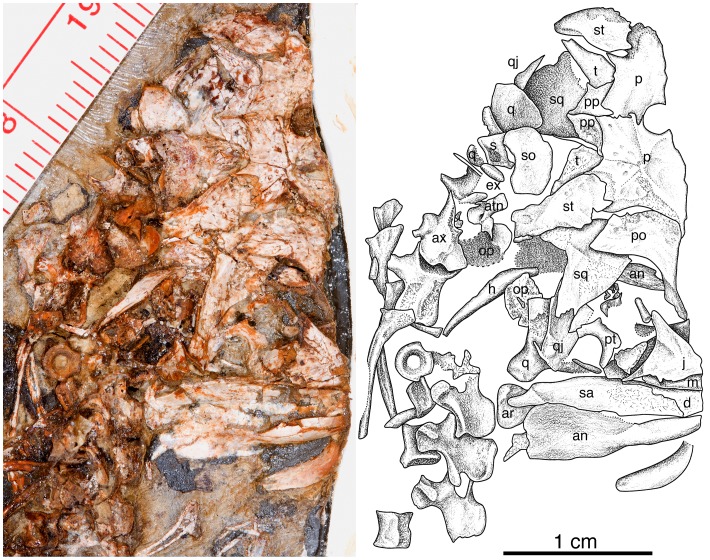
*Eocasea martini* gen. et sp. nov. Photograph and interpretive drawing of cranial anatomy of KUVP 9616b. Part of the skull table and cheeks, parts of occiput and braincase, and the posterior portion of the right mandible are preserved. Abbreviations: an, angular; ar, articular; atn, atlantal neural arch; ax, axis; d, dentary; ex, exoccipital; h, hyoid; j, jugal; m, maxilla; op, opisthotic; p, parietal, po, postorbital; pp, postparietal; pt, pterygoid; q, quadrate; qj, quadratojugal; s, stapes; sa, surangular; so, supraoccipital; sq, squamosal; st, supratemporal; t, tabular.

**Figure 3:**
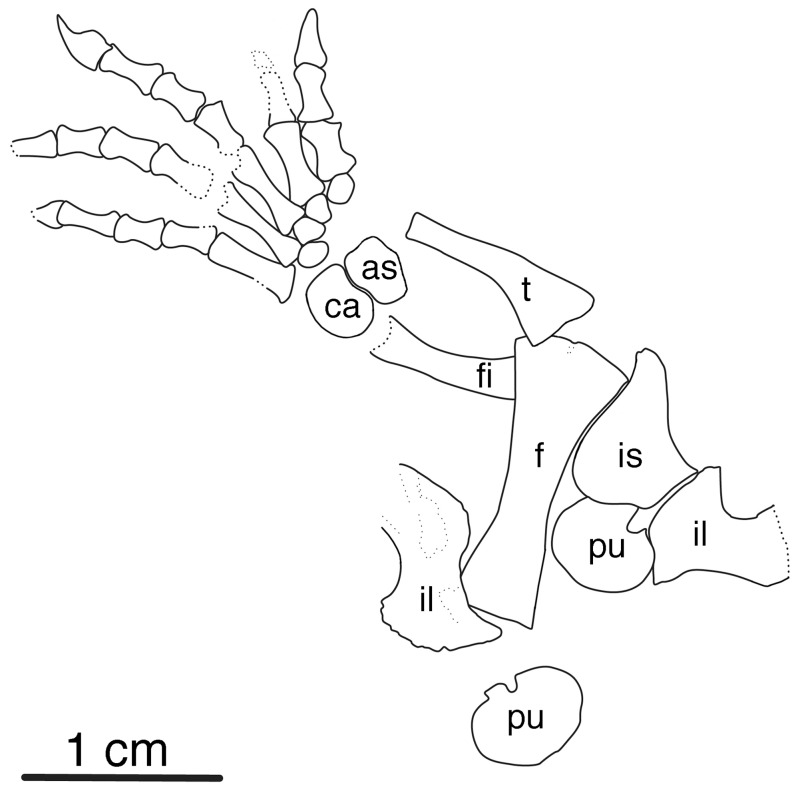
*Eocasea martini* gen. et sp. nov. Interpretive illustration of pelvic girdle and right hind limb of KUVP 9616b, drawn from reverse side through clear plastic embedding of specimen. Abbreviations: as, astragalus; ca, calcaneum; f, femur; fi, fibula; il, ilium; is, ischium; pu, pubis; t, tibia.

**Figure 4:**
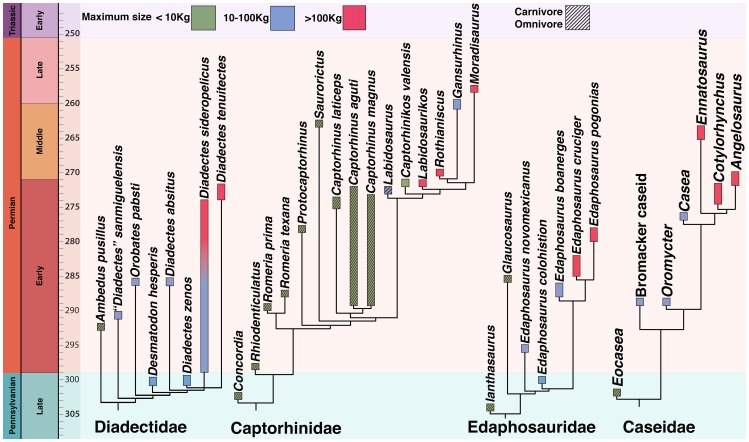
Time-calibrated phylogenies of the major lineages of early terrestrial tetrapod herbivores (Captorhinidae, Diadectidae, Caseidae, and Edaphosauridae). Estimates of maximum body size of included taxa are placed within three weight categories in order to indicate the general trends, less than 10(green), 10–100 kg (blue), and more than 100 kg (red). We restricted our size ranges to the above bins because several taxa are known only from fragmentary remains or juveniles. Only those herbivorous taxa that were sufficiently complete to permit size estimates were included. See Material and Methods for details on body mass estimates. The taxa shown as open, colored boxes of various shades of green are all considered to be herbivores. The taxa shown as crosshatched boxes are all considered to be either carnivorous, or omnivorous.

Here we describe a new small caseid (Figures 1, 2, 3) from the late Pennsylvanian of Kansas, USA, much older than any previously known member of this clade, and consider the significance of this discovery within the context of caseid phylogeny, and within the larger context of the origin and initial evolutionary history of herbivory in terrestrial vertebrates. We explore questions related to this major event in terrestrial vertebrate evolution within the framework of particular clades of herbivores, place the oldest known herbivores and their nearest non-herbivorous relatives into temporally calibrated phylogenies (Figure 4), for the first detailed examination of the mode and tempo of evolution of this novel feeding strategy.

## Materials and Methods

### Fossil Preparation

The holotype of *Eocasea martini* was originally collected by Dr. Larry Martin, the curator of the Dyke Museum of Natural History, University of Kansas, with permission from the owner of the locality, the Hamilton Quarry. No permit was required, and the specimen was curated and became part of the vertebrate paleontology collection of the above museum. The specimen was subsequently borrowed by the first author, with permission to prepare and study it. Most of its exposed bone surfaces were damaged. It was therefore necessary to imbed the skeleton in an epoxy resin and prepare it from the opposite side. This was done with the permission from the curator of the Dyke Museum. The preparation was performed through the use of 10% acetic acid, and manual preparation. This process resulted in exposure of much valuable anatomical information. However, during the initial process of acid preparation, the simple, conical dentition from the posterior part of the maxilla was lost.

### Body Size Estimates

Approximate weight estimates as indicators of overall size, as shown in Figure 4, were compiled using various sources. Size and weight measurements from Table 5, Romer and Price [21] were used as the principal source for the caseid and edaphosaurid weight estimates in Figure 4. The available synapsid data were augmented with new information from other sources [7], [11], [20], [22], [23]. Direct specimen measurements were made of some caseid taxa, like *Casea broilii*, and *Cotylorhynchus romeri* in order to find the largest known individuals (Appendix S1). When only partial skeletons were available for measurements, scaling was used to determine the approximate weight of that individual. Thus, the estimated weight measurements are largely comparative. For the purpose of this study we therefore used only three body weight ranges, less than 10 kg, greater than 10 kg, but less than 100 kg, and more than 100 kg. Weight estimates for the diadectids were obtained with assistance from Dr. Richard Kissel. Body estimates for the smaller taxa shown, shown in green as being less than 10 kg, took into account that they are juveniles (*Eocasea*, *Ianthasaurus*), or fragmentary (*Glaucosaurus, Ambedus*). Clearly, the adult members of *Eocasea* and *Ianthasaurus* would have been well below 10 kg in weight, but we cannot provide a reliable weight estimate. Similarly, the skull material of the fragmentary taxa like *Glaucosaurus* and *Ambedus* indicate that they were small animals, well below 10 kg in total body weight, but a more precise estimate was not possible. Comparisons between the use of vertebral measurements, as applied by Romer and Price [21] for their body mass estimates, and proximal limb bone dimensions in quadrupedal terrestrial vertebrates [24] using scaling relationships, have yielded surprisingly similar results for the caseid taxa, well within a 25% error range.

### Nomenclatural Acts

The electronic edition of this article conforms to the requirements of the amended International Code of Zoological Nomenclature, and hence the new names contained herein are available under that Code from the electronic edition of this article. This published work and the nomenclatural acts it contains have been registered in ZooBank, the online registration system for the ICZN. The ZooBank LSIDs (Life Science Identifiers) can be resolved and the associated information viewed through any standard web browser by appending the LSID to the prefix “http://zoobank.org/”. The LSID for this publication is: urn:lsid:zoobank.org:pub: 3641FFD8-FB41-4E5F-81FF-5E3D9AF42B1E. The electronic edition of this work was published in a journal with an ISSN, and has been archived and is available from the following digital repositories: PubMed Central, LOCKSS.

## Results

### Systematic PALEONTOLOGY

Synapsida Osborn, 1903 [25].

Caseasauria Williston, 1912 [26].

Caseidae Williston, 1911 [27].

*Eocasea martini* gen. et sp. nov.

urn:lsid:zoobank.org:act:D81C4A21-EC45-42FF-B4D7-ED79A5130BD8; 0C30568F-331D-40C6-A4C3-D9CBD0058B97.

### Etymology

The generic name refers to the fact that this is the oldest known member of the clade; the specific epithet is an homage to the late Dr. Larry Martin, the original collector of the holotypic specimen.

### Holotype

KUVP 9616b (Dyke Museum of Natural History, University of Kansas), a partial skull and postcranial skeleton of a juvenile individual, including the posterior one-third of the skull and right mandible, nearly complete vertebral column including cervical, dorsal, sacral and caudal vertebrae, pelvic girdle and nearly complete right hind limb (Figures 1, 2, 3).

No permits were required for the described study, which complied with all relevant regulations.

### Locality and Horizon

Hamilton Quarry near Hamilton, Greenwood County, Kansas, USA; Calhoun Shale, Shawnee Group, Virgilian Series (Stephanian of Europe), Upper Pennsylvanian.

### Diagnosis

A small caseid with the following autapomorphies: small, blade-like neural spines in dorsal and anterior caudal vertebrae; first sacral rib with very broad distal head, three times the size of that of the second sacral rib. Differs from other caseids in having unexpanded rib cage, last two dorsal ribs more slender than the other dorsals.

### Description

The cranial morphology of *Eocasea* provides clear and unequivocal evidence that this taxon is a caseasaurian, and a member of Caseidae. It has a large lateral temporal fenestra that is bordered by a posteroventrally narrow squamosal, and a large postorbital with a wide dorsal surface that contributes significantly to the skull table. As in all caseasaurians, the posterolateral wing of the parietal bone is broad and carries a large, wide supratemporal in a shallow groove on its dorsal surface. As in caseids, however, the supratemporal bone is not only very broad anteriorly, even broader than in the non-caseid caseasaurians *Eothyris* and *Oedaleops* [28], but also extends far posteriorly and ventrally, well beyond the posterior confines of the skull. In all other synapsids, including *Eothyris* and *Oedaleops,* the posteroventral extent of the supratemporal matches that of the tabular. However, in *Eocasea* and other caseids, the posterolateral wing of the tabular is relatively short, and does not reach the tip of the supratemporal. The pattern of sculpturing of the skull roof, with a relatively random distribution of rounded pits is only found in caseids. The morphology of the lateral temporal fenestra, although incompletely preserved, is sufficient to determine that the quadratojugal contributes to the ventral border of the opening, a feature that characterizes caseids. In contrast, a long posterior process of the jugal contacts the squamosal in *Eothyris* and *Oedaleops.* Although the preserved fragment of the maxilla is largely uninformative, this is partly a consequence of the initial process of acid preparation, as the delicate, simple, conical teeth from the posterior end of the maxilla were lost. The unprepared specimen originally showed the presence of simple conical dentition, with no evidence of any caseid type of serrations on the crown, or even the kind of gentle mesio-distal expansion of the crown present in *Oromycter* [9], [20].

The lower jaw is only partially preserved and exposed in lateral view. The posterior part of the dentary is exposed in lateral view, its dorsal edge being covered by the maxilla. It has a long posterior process that extends along the dorsal edge of the lower jaw, and covers part of the surangular in the region of the low coronoid eminence. In this regard, *Eocasea* exhibits the plesiomorphic condition, since the dorsal edge of the coronoid eminence is occupied by the coronoid and the surangular in other caseids. The angular, although somewhat flattened by crushing, appears to be taller than the surangular, a condition more reminiscent of *Eothyris* than *Cotylorhynchus.* The articular bone is a small bone at the posterior end of the mandible, and lacks a retroarticular process, as in all other caseids.

There are 27 presacral and two sacral vertebrae in *Eocasea,* a condition that is very close to the plesiomorphic condition (26 presacrals and two sacrals) for synapsids and all amniotes [7], [29]. Other caseids have a reduced number of presacrals (24–26) and higher number of sacrals (3–4). The axial neural arch is slightly damaged, but there is enough information to indicate that the neural spine is only slightly taller than in subsequent vertebrae, and leans slightly anteriorly, as in other caseids. Cervical vertebrae are similar in length to the dorsal vertebrae. Throughout the cervical and presacral part of the column, the vertebrae have simple, blade-like neural spines that are anteroposteriorly well developed, but relatively low in height. The caudal vertebrae of *Eocasea* have greatly reduced neural spines. All cervical, dorsal, and presacral vertebrae have relatively short transverse processes, in strong contrast to the other, herbivorous caseids, where the transverse processes extend far laterally. Many of the dorsal vertebrae have well-exposed, broad, relatively short and massive centra. There are no spaces ventrally for intercentra throughout much of the cervical and dorsal region, but three intercentra are present in the posterior region of the column, just anterior to the sacrum. As in other caseids, the centra have distinctive, flat ventral surfaces in the mid and posterior dorsal region, and are thus different from any other synapsids and reptiles.

In contrast to the condition in other caseids, the dorsal ribs of *Eocasea* are not thickened, and do not form a barrel-shaped, dorsolaterally expanded rib cage. In most caseids the lumbar ribs tend to fuse to the vertebrae, and remain massive and large, similar in cross section to the rest of the dorsal ribs, extending far laterally. In *Eocasea* the last three ribs, those just anterior to the sacrum, are small and more delicate than the other dorsal ribs, a condition that is similar to that seen in non-herbivorous synapsids. In contrast to other caseids, where there are either three or four sacral ribs, *Eocasea* has only two sacral ribs, with the first sacral being significantly broader distally than the second. The distal end of the second sacral rib is slightly expanded, and most likely extended to the iliac blade, just posterior to the first sacral. Five relatively massive caudal ribs are preserved on the right side of the skeleton, and there is a small transverse process on the sixth caudal vertebra for an additional rib. Hemal arches are associated with the 5^th^ caudal vertebra, and are present in all subsequent preserved caudals.

The shoulder girdle and forelimb are not preserved, but the pelvic girdle and the right hind limb are present. The ilium has the typical caseid morphology of a tall iliac blade with vertical grooves and ridges distally. The blade is centered directly above the acetabulum, with modest anterior and slightly larger posterior processes, similar to that seen in *Ruthenosaurus* [15] and most other caseids [22]. The right femur, tibia, and fibula are massively built, which is also consistent with caseid affinities. Beyond general outlines of the limbs, little detailed morphology of these limb elements are discernible because of the juvenile condition of the specimen. It was possible to reconstruct the outlines of the elements of the pes, even though it was partly covered by the caudal vertebrae because the skeleton was embedded into clear plastic in this area. In the tarsus, both the astragalus and calcaneum are preserved as relatively elongate elements, but of the tarsals only the centrale, or the fifth tarsal are ossified, indicating the specimen’s immature nature. The metatarsals are all preserved, and as expected in a caseid, the 1^st^ metatarsal is long and massive, nearly equal in length to the 4^th^ and 5^th^ in the series. This is in contrast to the condition in most other early synapsids, in which the 4^th^ metatarsal is by far the longest in the series, and significantly longer than the 1^st^. Similarly, all preserved phalanges are approximately equal in length, and the terminal, claw-bearing phalanx is large, with a massive adductor tubercle. The phalangeal formula in the pes of *Eocasea* is 2-3-4-5-4 like that in *Casea broilii,* representing the plesiomorphic condition for amniotes. All other caseids where the phalangeal count is known reduce the number of phalanges in the pes.

### Phylogenetic Analysis

*Eocasea* is clearly identifiable as a member of Caseidae, despite the absence of part of the skull, the shoulder girdle and forelimbs. In the skull, the large size and distinct morphology of the supratemporal, the differences in size between this element and the tabular, the unique sculpturing pattern, the reduced ventral process of the squamosal are all synapomorphies shared with other caseids. The absence of a sharp keel on the ventral surface of the vertebral centra precludes its assignment to the more derived eupelycosaurs such as varanopids and sphenacodontids [7], [21], [23]. In contrast, the distinct flat ventral surface of the vertebral centra is typical for caseids [15], [22]. The pelvis is also characteristic for a caseid with respect to the relative size and morphology of the ilium. The massiveness of the limbs and the proportions of the pes further support this interpretation.

A phylogenetic analysis using PAUP* 4.0b10 [30] was performed using a previously compiled data matrix [9], but incorporating *Eocasea* in order to evaluate its position among Caseasauria. 73 cranial and 33 postcranial characters were used in this analysis, yielding a single most-parsimonious tree with a length of 200 steps, with *Eocasea* positioned as a sister taxon to all other caseids. The list of characters, the character coding for *Eocasea,* and the PAUP results are included as Appendix S2, S3, S4). The tree has a Consistency index (CI) of 0.702, a Homoplasy Index (HI) of 0.298, a Retention Index (RI) of 0.7595, and a Rescaled Consistency Index (RC) of 0.543. Bootstrap and Bremer decay analyses yielded similar results to that of the previous analysis (with 91% and 2 for the node of *Eocasea*+other caseids, respectively).

In order to test the affinities of *Eocasea* within the Synapsida, we also included it in the most recent phylogeny that included a broad spectrum of early synapsids [31]. We performed this analysis, even though the first author discovered numerous errors and omissions in the character codings. A thorough review of early synapsid phylogeny is currently in progress, but the addition of *Eocasea* into this somewhat problematic data matrix, and the resulting phylogenetic analysis still positions *Eocasea* within Caseidae (Appendix S5). Interestingly, the inclusion of *Eocasea* in this phylogenetic analysis results in a tree topology that is consistent with previous studies [7], [13], [28], and different from those in the latest early synapsid phylogeny [31].

## Discussion

The discovery of *Eocasea* at the Hamilton Quarry Locality in southeastern Kansas, USA, is consistent with previous important discoveries of amniotes at this site. This locality, a Fossillagerstätte (a sedimentary deposit with extraordinary preservation of fossils) in a tidally influenced paleovalley setting, where rapid sediment deposition preserves an unusually diverse flora and fauna [32]. The vertebrate fauna is dominated by hundreds of superbly preserved acanthodian fish and numerous dissorophoid amphibians. Only a few amniotes, all small terrestrial carnivores of great evolutionary significance have been recovered [33]–[35]. The remains of all amniotes are represented by small skeletons, showing no evidence of predation or scavenging, but some evidence of flotation. The single known skeleton of *Eocasea* was probably washed into the paleovalley, and like the other small amniotes, was not a normal member of the aquatic vertebrate fauna. Previous significant finds include a single articulated skeleton of *Spinoaequalis,* the second oldest known diapsid reptile [32], a single articulated skeleton of *Archaeovenator,* the oldest known and most basal varanopid synapsid [33], and two specimens of *Concordia cunninghami*, the oldest known and most basal captorhinid reptile [34].

*Eocasea* changes significantly our understanding of the evolutionary history of both caseids and caseasaurs. The known fossil record of caseasaurs has been very disjointed, with eothyridids, the sister taxon of caseids being Asselian and Artinskian in age, and much older than most caseids which are restricted mainly to Kungurian age localities in North America, and younger, Middle Permian age localities in Europe. The discovery of *Eocasea* extends the fossil record of Caseasauria and Caseidae significantly, well into the Pennsylvanian, in line with the fossil record of other early synapsid clades, indicating that the initial stages of synapsid diversification were well under way by this time. More significantly, however, *Eocasea* also allows us to re-evaluate the origin and evolution of herbivory within this clade, and terrestrial vertebrates in general.

### Origin of Herbivory

Herbivory, as defined in this paper, is one of the most important innovations in terrestrial vertebrate evolution, and requires a number of anatomical and physiological attributes for efficient breakdown of the cellulose rich walls of terrestrial plants [1], [2]. Prominent among these, and paleontologically observable, are changes in the trunk. Cellulysis typically occurs in the digestive tract, and herbivorous tetrapods have longer and bulkier digestive tracts (and thus a longer and/or broader trunk region) than related carnivorous or insectivorous forms because part of the gut is modified to house vast numbers of endosymbionts for the fermentative breakdown of cellulose from the plant fodder [3]. Thus, we can identify Paleozoic herbivores because their rib cages are typically significantly wider and more capacious than those of their closest insectivorous or carnivorous relatives [1], [2]. Nevertheless, it is likely that the ability to process this kind of plant matter precedes the skeletal correlates that can be found in the fossil record. It is therefore possible that we are underestimating the extent of herbivory that existed in the Paleozoic, but this does not invalidate our results because the clades of herbivores that we examine here are widely separated by successive clades of non-herbivorous vertebrates [1], [2].

In the case of caseids, herbivory is indicated by the presence of a massive rib cage in the thoracic and dorsal regions, and the expanded trunk extends posteriorly to the pelvic girdle, with large ribs fused to the lumbar vertebrae. This osteological evidence of herbivory is present in all caseids, except for *Eocasea,* suggesting that this feeding strategy originated sometime between the late Pennsylvanian and the Early Permian. However, caseids are exceedingly rare in the first 25 million years of the Permian [36], with only two exceptions. Both occurrences are of Sakmarian age and in unusual, upland localities: *Oromycter* from the fissure fills near Richards Spur, Oklahoma, and a new, undescribed taxon from the Bromacker Quarry, Germany [19], [20]. Whereas other caseids also show dental specializations, with leaf-like large serrations being present in the marginal dentition, *Eocasea, Oromycter,* and the undescribed Bromacker Quarry caseid lack these serrations. Interestingly, both *Oromycter,* and the Bromacker caseid show skeletal evidence for herbivory, raising the possibility that oral processing in the form of puncturing vegetation may have evolved within Caseidae after the acquisition of herbivory.

The two localities that have yielded these early caseids are the only ones known to preserve upland faunas during the Early Permian, quite distinct from the typical, widely distributed deltaic, floodplain depositional environments that characterize the vast majority of the Early Permian fossil vertebrate localities of Pangaea, and that are completely devoid of caseid remains. Caseids become common elements of the lowland environments only in the latter part of the Early Permian, during the Kungurian. This occurrence pattern suggests that the early history of caseid evolution, and their initial acquisition of herbivory may have occurred in upland environments. In strong contrast, the early Permian lowland localities contain the remains of two other groups of herbivorous tetrapods, the diadectids and edaphosaurids.

The oldest known herbivorous edaphosaur is *Edaphosaurus novomexicanus* from the Permo-Carboniferous of New Mexico [12], while the slightly older *Ianthasaurus* from Kansas [11], [37], [38] is a small omnivore with a narrow rib cage. As in other, younger members of the genus *Edaphosaurus*, *E. novomexicanus* shows the rib modifications that characterize herbivores, but in contrast to caseids, it also has massive occluding dentition for extensive oral processing [2]. Herbivorous edaphosaurs and caseids do not overlap to any significant extent in the fossil record, with edaphosaurs being restricted to the latest Pennsylvanian and early part of the Early Permian (303-280 Ma), the fossil record of well known caseids being restricted to the later part of the Early Permian, and younger strata (275-260 Ma) [36]. They also represent different feeding strategies, caseids using little oral processing, edaphosaurs relying on extensive oral processing.

Two other groups of terrestrial vertebrates also acquire herbivory in this initial stage of amniote diversification, diadectid cotylosaurs and captorhinid reptiles. Late Pennsylvanian and Early Permian diadectids also show convincing evidence of dental and skeletal adaptations for herbivory. These enigmatic Paleozoic forms are part of Diadectomorpha, a sister group to crown Amniota. A preliminary phylogeny of diadectids indicates that *Ambedus,* a small diadectid from the Early Permian, tentatively identified as omnivorous because of its labiolingually expanded cheek teeth (but no evidence of dental wear) is the sister taxon to all other diadectids [6]. However, the oldest known diadectid from the late Pennsylvanian of Oklahoma is already clearly an herbivore [39] and older than the edaphosaur *Edaphosaurus novomexicanus*. As is the case with the caseid and edaphosaur synapsids, the sister taxon of Diadectidae, the Early Permian *Tseajaia* from New Mexico, was faunivorous.

The fossil record of captorhinids, the first group of reptiles to diversify extensively in the Paleozoic, extends from the late Pennsylvanian into the Late Permian. Despite an extensive, rich fossil record, evidence of herbivory appears late in the history of this group, in the uppermost part of the Kungurian [40], much later than in either diadectids or synapsids.

An enigmatic group of early parareptiles, the bolosaurids, also show evidence of herbivory. The fossil record of these poorly known small parareptiles extends from the earliest Permian of New Mexico to the Middle Permian of China [41], [42]. All the known members of the clade are characterized by occluding cheek dentition, with wear facets that suggest that these small reptiles were herbivores. Since the known skeletal remains of bolosaurids are almost entirely restricted to jaws and teeth, we can identify only those members of this clade that have the specialized dentition for herbivory. In fact, little is known about the overall cranial anatomy of most bolosaurids, with the exception of *Belebey,* and *Eudibamus* [41], and only the postcranial anatomy of *Eudibamus* [42] has been described. Overall, there is little information on the evolutionary history of bolosaurids, making any detailed consideration of the origin and evolution of herbivory in this group impossible. If they are truly herbivores, as their dentition suggests, bolosaurids represent yet another example of the independent acquisition of this feeding strategy at this stage of terrestrial vertebrate evolution because this clade of small reptiles is deeply nested among non-herbivorous parareptiles [1].

The overall pattern provided by the fossil record of synapsids, diadectids, and captorhinids (and possibly bolosaurids) allows us to conclude that herbivory evolved independently in these clades for the first time in the late Pennsylvanian at a time when the terrestrial flora was undergoing some major changes [43], [44]. The fossil record indicates that acquisition of herbivory in the four clades was staggered, but the overall pattern is that once this ability to process high fiber plant material evolved in terrestrial vertebrates, at least four separate lineages acquired it independently within the late Pennsylvanian and Early Permian, by using different feeding strategies. How this apparent, major evolutionary threshold was breached near the end of the Pennsylvanian remains a mystery at this time, but once acquired by these terrestrial vertebrates, the pattern of multiple, independent evolution of herbivores occurs repeatedly and almost continuously in the fossil record [43], [44]. It is therefore particularly important to study and evaluate the evolution of herbivory during this first initial stage, when the modern terrestrial ecosystem was not yet established.

### Body Size in Early Herbivores

Direct comparisons between time-calibrated phylogenies (Figure 4) of caseids, edaphosaurids, diadectids and captorhinids [11], [45]–[48] reveal that herbivory is consistently correlated with substantial increase in body size, with the most basal, non-herbivorous members of each clade being small, always less than 10 kg in estimated weight (Figure 4). This pattern is most striking among caseids (Appendix S1). Although the holotype of *Eocasea* certainly represents a juvenile individual, it is diminutive, with an estimated snout-vent length of 125 mm (Appendix S6). In contrast, the smallest known herbivorous caseid with a comparable ontogenetic age, based on level of ossification of the vertebrae and pedal elements, is a basal, undescribed form from Germany [19] and has an estimated snout-vent length of 400 mm. The adult skeleton of the same species has a snout-vent length of about 700 mm (Reisz, pers. obs.). Estimates of body size [21] indicate that caseids range in size from an estimated weight of less than 10 kg in *Eocasea* to more than 500 kg in the largest known specimen of *Cotylorhynchus hancocki* [22].

A somewhat similar pattern is also present in some of the carnivorous clades of synapsids, with early or basal members of each clade being smaller than the younger, more derived species, but carnivorous clades do not show the dramatic increases in size seen in the herbivorous clades. For example, among varanopids, a clade of agile predators that have a similar temporal range as the caseids, we see an increase in size from less than 10 kg in *Archaeovenator* [34] to a maximum of about 50 kg in the largest and youngest member of the clade, *Watongia* [47]. Among the top predators, the most dramatic size range is seen among the sphenacodontid synapsids, with the maximum body size ranging from 52 kg in *Sphenacodon ferox* to 254 kg in *Dimetrodon grandis* within the Early Permian [21].

## Conclusions

*Eocasea martini* represents the earliest and most basal known caseid synapsid, extending the fossil record of Caseasauria into the Pennsylvanian. Its discovery fills a significant gap in the fossil record, indicating that this important clade of early synapsids is much more ancient than previously documented in the fossil record. *Eocasea* provides clear evidence that large caseid herbivores, the largest known terrestrial vertebrates of their time, evolved from small non-herbivorous members of that clade. This pattern is mirrored by several other Permo-Carboniferous clades that include early herbivores (Diadectidae, Edaphosauridae, and Captorhinidae). Among amniotes, it is the synapsids, on the mammalian rather than the reptilian side of higher vertebrate evolution, that were able to acquire herbivory early in their evolutionary history. The available evidence indicates that this innovation in feeding behavior led by the end of the Early Permian to the establishment of a modern type of trophic structure in terrestrial vertebrate ecosystems, one in which numerous herbivores support relatively few top predators [2], [49]. Significantly, it is synapsids that evolved first both herbivores and large terrestrial predators in the evolutionary history of terrestrial vertebrates, a pattern that was maintained until the end of the Paleozoic.

## Supporting Information

Appendix S1 **Specimen measurements and weight estimates.**
(PDF)

Appendix S2 **Caseid Character list used in phylogenetic analysis+** ***Eocasea*** **coding.**
(PDF)

Appendix S3 **Caseid Phylogenetic Analysis: PAUP results.**
(PDF)

Appendix S4 **Data Matrix of** ***Eocasea+*** **other caseids.**
(NEX)

Appendix S5 **Character coding and PAUP results based on Benson 2012+** ***Eocasea.***
(PDF)

Appendix S6 ***Eocasea*** **specimen measurements.**
(PDF)

## References

[1] Reisz RR, Sues HD (2000) Herbivory in late Paleozoic and Triassic terrestrial vertebrates. In: Sues HD, editor. Evolution of Herbivory in Terrestrial Vertebrates: Perspectives from the Fossil Record. Cambridge: Cambridge University Press. pp. 9–41.

[2] Sues H-D, Reisz RR (1998) Origins and early evolution of herbivory in tetrapods. Trends in Ecology & Evolution 13: 141–145. 21238234 10.1016/s0169-5347(97)01257-3

[3] O’Grady PO, Morando M, Avila L, Dearing DM (2005) Correlating diet and digestive tract specialization: Examples from the lizard family Liolaemidae. Zoology 108: 201–210. 16351968 10.1016/j.zool.2005.06.002

[4] Barrett PM (2000) Prosauropod dinosaurs and iguanas: speculations on the diets of extinct reptiles. In: Sues HD, editor. Evolution of Herbivory in Terrestrial Vertebrates. Cambridge: Cambridge University Press. pp. 42–78.

[5] Smithson TR, Wood SP, Marshall JEA, Clack JA (2012) Earliest Carboniferous tetrapod and arthropod faunas from Scotland populate Romer’s Gap. Proceedings of the National Academy of Sciences 109: 4532–4537. 10.1073/pnas.1117332109 PMC3311392 22393016

[6] Kissel RA, Reisz RR (2004) Synapsid fauna of the Upper Pennsylvanian Rock Lake Shale near Garnett, Kansas and the diversity pattern of early amniotes. In: Arratia G, Wilson MVH, Cloutier R, editors. Recent Advances in the Origin and Early Radiation of Vertebrates. München: Pfeil Verlag. 409–428.

[7] Reisz RR (1986) Pelycosauria. Stuttgart: G. Fischer. 102 p.

[8] Reisz RR (1997) The origin and early evolutionary history of amniotes. Trends in Ecology & Evolution 12: 218–222. 21238045 10.1016/s0169-5347(97)01060-4

[9] Maddin HC, Sidor CA, Reisz RR (2008) Cranial anatomy of *Ennatosaurus tecton* (Synapsida : Caseidae) from the middle permian of Russia and the evolutionary relationships of Caseidae. Journal of Vertebrate Paleontology 28: 160–180.

[10] Modesto SP (1995) The skull of the herbivorous synapsid *Edaphosaurus boanerges* from the Lower Permian of Texas. Palaeontology 38: 213–239.

[11] Mazierski DM, Reisz RR (2010) Description of a new specimen of *Ianthasaurus hardestiorum* (Eupelycosauria: Edaphosauridae) and a re-evaluation of edaphosaurid phylogeny. Canadian Journal of Earth Sciences 47: 901–912.

[12] Modesto SP, Reisz RR (1992) Restudy of Permo-Carboniferous synapsid *Edaphosaurus novomexicanus* Williston and Case, the oldest known herbivorous amniote. Canadian Journal of Earth Sciences 29: 2653–2662.

[13] Dilkes DW, Reisz RR (1996) First record of a basal synapsid (‘mammal-like reptile’) in Gondwana. Proceedings of the Royal Society of London Series B-Biological Sciences 263: 1165–1170.

[14] Reisz RR, Laurin M (2001) The reptile *Macroleter*: First vertebrate evidence for correlation of Upper Permian continental strata of North America and Russia. Geological Society of America Bulletin 113: 1229–1233.

[15] Reisz RR, Maddin HC, Fröbisch J, Falconnet J (2011) A new large caseid (Synapsida, Caseasauria) from the Permian of Rodez (France), including a reappraisal of *“Casea” rutena* Sigogneau-Russell & Russell, 1974. Geodiversitas 33: 227–246.

[16] Schneider JW, Koerner F, Roscher M, Kroner U (2006) Permian climate development in the northern peri-Tethys area - The Lodeve basin, French Massif Central, compared in a European and global context. Palaeogeography Palaeoclimatology Palaeoecology 240: 161–183.

[17] Lopez M, Gand G, Garric J, Körner F, Schneider JW (2008) The playa environments of the Lodève Permian basin (Languedoc-France). Journal of Iberian Geology 34: 29–56.

[18] Harris SK, Lucas SG, Berman DS, Henrici AC (2004) Vertebrate fossil assemblage from the Upper Pennsylvanian Red tanks member of the Bursum Formation, Lucero uplift, central New Mexico. Bulletin of the New Mexico Museum of Natural History and Science 25: 267–284.

[19] Berman D, Henrici A, Sumida S (2009) Pelycosaurian-grade synapsids from the Lower Permian Bromacker locality, central Europe. Journal of Vertebrate Paleontology 29: 62A–62A.

[20] Reisz RR (2005) *Oromycter*, a new caseid from the Lower Permian of Oklahoma. Journal of Vertebrate Paleontology 25: 905–910.

[21] Romer AS, Price LI (1940) Review of the Pelycosauria. Geological Society of America Special Paper 28: 1–538.

[22] Olson EC (1968) The family Caseidae. Fieldiana 17: 225–349.

[23] Stovall JW, Price LI, Romer AS (1966) The postcranial skeleton of the giant Permian pelycosaur *Cotylorhynchus romeri* . Bulletin of the Museum of Comparative Zoology 135: 1–30.

[24] Campione N, Evans D (2012) A universal scaling relationship between body mass and proximal limb bone dimensions in quadrupedal terrestrial tetrapods. BMC Biology 10: 60. 22781121 10.1186/1741-7007-10-60 PMC3403949

[25] Osborn HF (1903) On the primary division of the Reptilia into two sub-classes Synapsida and Diapsida. Science 17: 275–276. 10.1126/science.17.424.275-b 17740617

[26] Williston SW (1912) Primitive reptiles. A review. Journal of Morphology 23: 637–666.

[27] Williston SW (1911) Permian reptiles. Science 33: 631–632. 17798180 10.1126/science.33.851.631

[28] Reisz RR, Godfrey SJ, Scott D (2009) *Eothyris* and *Oedaleops*: do these early Permian synapsids from Texas and New Mexico form a clade? Journal of Vertebrate Paleontology 29: 39–47.

[29] Müller J, Scheyer TM, Head JJ, Barrett PM, Werneburg I, et al (2010) Homeotic effects, somitogenesis and the evolution of vertebral numbers in recent and fossil amniotes. Proceedings of the National Academy of Sciences of the United States of America 107: 2118–2123. 20080660 10.1073/pnas.0912622107 PMC2836685

[30] Swofford DL (2001) PAUP*: phylogenetic analysis using parsimony (*and other methods). Sunderland, MA: Sinauer Associates.

[31] Benson RBJ (2012) Interrelationships of basal synapsids: cranial and postcranial morphological partitions suggest different topologies. Journal of Systematic Palaeontology 10: 601–624.

[32] Cunningham CR, Feldman HR, Franseen EK, Gastaldo RA, Mapes G, et al (1993) The Upper Carboniferous Hamilton Fossil-Lagerstatte in Kansas: a valley-fill, tidally influenced deposit. Lethaia 26: 225–236.

[33] deBraga M, Reisz RR (1995) A new diapsid reptile from the uppermost Carboniferous (Stephanian) of Kansas. Palaeontology 38: 199–212.

[34] Reisz RR, Dilkes DW (2003) *Archaeovenator hamiltonensis*, a new varanopid (Synapsida : Eupelycosauria) from the Upper Carboniferous of Kansas. Canadian Journal of Earth Sciences 40: 667–678.

[35] Müller J, Reisz RR (2005) An early captorhinid reptile (Amniota, Eureptilia) from the Upper Carboniferous of Hamilton, Kansas. Journal of Vertebrate Paleontology 25: 561–568.

[36] Brocklehurst N, Kammerer CF, Fröbisch J (2013) The early evolution of synapsids, and the influence of sampling on their fossil record. Paleobiology 39: 470–490.

[37] Modesto SP, Reisz RR (1990) A new skeleton of *Ianthasaurus hardestii*, a primitive edaphosaur (Synapsida, Pelycosauria) from the Upper Pennsylvanian of Kansas. Canadian Journal of Earth Sciences 27: 834–844.

[38] Reisz RR, Berman DS (1986) *Ianthasaurus hardestii* n. sp., a primitive edaphosaur (Reptilia, Pelycosauria) from the Upper Pennsylvanian Rock Lake Shale near Garnett, Kansas. Canadian Journal of Earth Sciences 23: 77–91.

[39] Kissel RA, Lehman TM (2002) Upper Pennsylvanian tetrapods from the Ada Formation of Seminole County, Oklahoma. Journal of Paleontology 76: 529–545.

[40] Dodick JT, Modesto SP (1995) The cranial anatomy of the captorhinid reptile *Labidosaurikos meachami* from the Lower Permian of Oklahoma. Palaeontology 38: 687–711.

[41] Reisz RR, Müller J, Tsuji L, Scott D (2007) The cranial osteology of *Belebey vegrandis* (Parareptilia: Bolosauridae), from the Middle Permian of Russia, and its bearing on reptilian evolution. Zoological Journal of the Linnean Society 151: 191–214.

[42] Berman D S, Reisz RR, Henrici AC, Sumida SS, Martens T (2000) Early Permian Bipedal Reptile. Science 290: 969–972. 11062126 10.1126/science.290.5493.969

[43] Sahney S, Benton MJ, Falcon-Lang HJ (2010) Rainforest collapse triggered Carboniferous tetrapod diversification in Euramerica. Geology 38: 1079–1082.

[44] Pearson MR, Benson RBJ, Upchurch P, Fröbisch J, Kammerer CF (2013) Reconstructing the diversity of early terrestrial herbivorous tetrapods. Palaeogeography, Palaeoclimatology, Palaeoecology 372: 42–49.

[45] Kissel RA, Reisz RR (2004) *Ambedus pusillus*, new genus, new species, a small diadectid (Tetrapoda : Diadectomorpha) from the Lower Permian of Ohio, with a consideration of diadectomorph phylogeny. Annals of Carnegie Museum 73: 197–212.

[46] Kissel RA, Reisz RR, Berman DS (2005) Revisiting the taxonomy of Diadectidae (Cotylosauria: Diadectomorpha): a phylogenetic approach. Journal of Vertebrate Paleontology 25: 78A.

[47] Reisz RR, Laurin M (2004) A reevaluation of the enigmatic Permian synapsid *Watongia* and of its stratigraphic significance. Canadian Journal of Earth Sciences 41: 377–386.

[48] Reisz RR, Liu J, Li J-L, Müller J (2011) A new captorhinid reptile, *Gansurhinus qingtoushanensis*, gen. et sp. nov. from the Permian of China. Naturwissenschaften 98: 435–441. 21484260 10.1007/s00114-011-0793-0

[49] Olson EC (1966) Community evolution and the origin of mammals. Ecology 47: 291–302.

