# Supplementary material for: The Oldest Caseid Synapsid from the Late Pennsylvanian of Kansas, and the Evolution of Herbivory in Terrestrial Vertebrates
Source: PLoS One. 2014 Apr 16;9(4):e94518. doi: 10.1371/journal.pone.0094518 (PMC3989228; doi:10.1371/journal.pone.0094518)
Supplement: Appendix S1 — Specimen measurements and weight estimates. (PDF) [file pone.0094518.s001.pdf]

Museum & Specimen	taxon	taxon group	femur lgth	femur circ	humerus lgth	humerus circ	skull length	max.length	vert. length	weight estimate
(Field Mus) UC1002	*Dimetrodon grandis*	sphenacodontid	260	115	220	128	482	200	45	>100 (265)
(Oklahoma Mus) OM-S4/S5	*Cotylorhynchus romeri*	caseid	308		338		171	80	47	>100 (331)
(Field Mus) UR581	*Cotylorhynchus hancocki*	caseid	392	220	410	216			68	>100 (>350)
(Field Mus) UR835	*Cotylorhynchus bransoni*	caseid	254	116					37	>100 (>280)
(Field Mus) UR257	*Angelosaurus greeni*	caseid	261	128						>100 (>280)
(Field Mus) UR149	*Angelosaurus dolani*	caseid	213	130	189	205			39	>100 (>250)
(Field Mus) UC656	*Casea br*	caseid	76	31.5	86	40	84	40	13	10-100 (21)
(Dyke Mus) KUVP	*Eocasea*	caseid	15	5.2					2.9	<10 (2)
(Russian Acad. Sci) PIN 4531/1	*Ennatosaurus*	caseid					165	78		>100 (280)
(Field Mus) FMNH 2281	*Oromycter*	caseid						42	14	10-100 (>25)
(Gotha.Ger) MNG 13814	*Bromacker caseid*	caseid	83		88		78	42	14	10-100 (>25)
(Field Mus) WM658	*Edaphosaurus cruciger*	edaphosaurid					157		41	>100 (166)
(Field Mus) WM239	*Edaphosaurus pogonias*	edaphosaurid	215		197		212		46	>100 (186)
(UofMichigan)UM1165	*Edaphosaurus boanerges*	edaphosaurid	176		162		140		34	10-100 (85)
(Field Mus)UC 691	*Glaucosaurus*	edaphosaurid					49	27		10-100
(Dyke Mus) KUVP 69035	*Ianthasaurus*	edaphosaurid			48		82	45	8.5	10-100
(Nat.Mus.Paris) MOR72	*Moradisaurus*	captorhinid					410	220		>100
(Chinese Acad. Sci)IVPP V331	*Gansurhinus*	captorhinid						62		10-100
(Field Mus) CNHM 87/131	*Rothianiscus*	captorhinid					265		16	>100
(Oklahoma Mus)OMNH 04331	*Labidosaurikos*	captorhinid					280	132		>100
(Carnegie Mus) CNMH 73371	*Labidosaurus*	captorhinid	83		75		198	84	13	10-100
(Oklahoma Mus) OMNH 55386-87	*Captohinus magnus*	captorhinid	43		41		78	48		<10
(Univ. Calif) UCLA 3214	*Captorhinus aguti*	captorhinid	36		31		58	27	8	<10
(Iziko Mus) SAM PK8666	*Saurorictus*	captorhinid					24	7.8		<10
(Univ. Harvard) MCZ1478	*Protocaptorhinus*	captorhinid			23		50	28		<10
(Univ. Harvard) MCZ1480	*Romeria texana*	captorhinid					54	29		<10
(Univ. Harvard) MCZ 1963	*Romeria prima*	captorhinid					53	28		<10
(Univ. Calif) UCMP35757	*Rhiodenticulatus*	captorhinid	16.6		15.6		42	18.3	3.8	<10
(Dyke Mus) KUVP8702	*Concordia*	captorhinid					38	15		<10
(Amer.Mus) AMNH 4375/MCZ 7850	*Diadectes tenuitectes*	diadectid	275				284	150		>100
(Univ. Harvard) MCZ1717	*Diadectes sideropelicus*	diadectid	200							>100
(Field Mus) UC675	*Diadectes sideropelicus*	diadectid					195	80		>100
(Field Mus) UC 679	*Diadectes zenos*	diadectid	146		144		167			<100
(Gotha.Ger) MNG8853	*Diadectes absitus*	diadectid	91		120		140	65		10-100
(Carnegie Mus) CM47654	*Desmatodon hesperis*	diadectid					148	60		10-100
(Gotha.Ger) MNG10181	*Orobates*	diadectid	77		75		130	58	13	10-100
(Univ. Harvard) MCZ2989	*"Diadectes" sanmiguelensis*	diadectid					100	30		10-100
(Univ. Harvard) MCZ9436	*Ambedus*	diadectid						18		<10
