# Supplementary material for: The Oldest Caseid Synapsid from the Late Pennsylvanian of Kansas, and the Evolution of Herbivory in Terrestrial Vertebrates
Source: PLoS One. 2014 Apr 16;9(4):e94518. doi: 10.1371/journal.pone.0094518 (PMC3989228; doi:10.1371/journal.pone.0094518)
Supplement: Appendix S2 — Caseid Character list used in phylogenetic analysis+ Eocasea coding. (PDF) [file pone.0094518.s002.pdf]

Data for phylogenetic analysis

A) DESCRIPTION OF CHARACTERS USED IN PHYLOGENETIC ANALYSIS. LISTED AS IN MADDIN ET AL, 2008 [7], WITH SLIGHT MODIFICATIONS SHOWN IN BOLD.

SKULL ROOF

1) Shape of premaxillary ascending process: slopes posterodorsally from tooth row (0); projects anterodorsally to overhang tooth row (1).

2) Shape of the antorbital region: maxilla slopes dorsolaterally, overhanging tooth row (0); maxilla slopes dorsomedially (1).

3) Subnarial foramina on dorsal edge of maxilla: small or absent (0); present and large (1).

4) External naris length: small, less than 25% preorbital length (0); moderate, 25–50% preorbital length (1); long, greater than 50% preorbital length (2).

5) External naris height: 30–75% of orbital height (0); greater than 75% or equal to orbital height (1).

6) Contribution of nasal to external naris: confined dorsally (0); bladelike, anteroventral portion of narial emargination (1).

7) Medial flange on maxilla forming vertical wall emarginating external naris: absent (0); present (1).

8) Postnarial ascending process of maxilla: absent (0); present and rounded dorsally (1); present and pointed dorsally (2).

9) Position of apex of postnarial ascending process of maxilla: process absent (0); tallest point located centrally (1); tallest point located anteriorly (2).

10) Length of posterior ramus of maxilla: confined to suborbital region (0); continues posteriorly beyond orbital region (1).

11) Dentition on posterior ramus of maxilla: present to posterior margin of orbital exclusively (0); terminates under postorbital bar (1); continues posterior to postorbital bar (2).

12) Shape of ventral edge of maxilla: straight (0); mildly to strongly convex (1).

13) Maxilla-quadratojugal contact: absent, jugal reaches ventral margin of skull in lateral view (0); present, jugal excluded from ventral margin of skull in lateral view (1).

14) Postparietal contribution of margin of posttemporal fenestra: absent (0); present (1).

15) Postorbital contribution to skull table: large, wide (0); small, narrow (1).

16) Shape of lacrimal: dorsoventral dimension less than length (0); dorsoventral dimension greater than length (1).

17) Lacrimal contribution to medial emargination of external naris: absent (0); present (1).

18) Jugal-squamosal contact below level of orbit: absent (0); present (1).

19) Morphology of anterior ramus of jugal: thin, sliver-like (0); thick and robust (1).

20) Height of dorsal ramus of jugal: less than 50% height of orbit and broad (0); greater than or equal to 50% height of orbit and slender (1).

21) Height of lateral temporal fenestra: lateral temporal fenestra absent (0); short, temporal fenestra height to temporal roof height ratio 0.5 or less (1); tall, temporal fenestra height to temporal roof height ratio 0.575 or greater (2).

22) Postorbital shape: smoothly rounded between skull table and temporal portions (0); right angle formed between skull table and temporal portions (1).

23) Extent of the contribution of frontal to orbital rim: none or small, less than 1/10 maximum orbital length (0); large, more than 1/5 maximum orbital length (1).

24) Size of pineal foramen: maximum diameter less than that of foramen magnum (0); maximum diameter equal to or greater than that of foramen magnum (1).

25) Frontal:parietal ratio of lengths at midline: frontal less than 1.5 times parietal length (0); frontal greater than 1.6 times parietal length (1).

26) Position of pineal foramen on parietal suture: anteriorly located on parietal suture (0); centrally located on suture

(1); near posterior of parietal suture (2).

27) Postorbital-supratemporal contact: present (0); absent, postorbital reaching level of supratemporal (1); absent, postorbital and supratemporal widely separated (2).

28) Supratemporal shape: broad, subrectangular, superficial bone on skull roof, length less than that of parietal at midline (0); small splint-like element in groove in parietal (1); large, elongate, roughly equal in length to parietal at midline (2).

29) Length of lateral temporal fenestra: absent (0); moderate, between 15–25% of total skull length (1); large, greater than 30% of total skull length (2).

PALATE

30) Dentition on cultriform process of parasphenoid: absent (0); present (1).

31) Parasphenoid body shape: wide, maximum width greater than length from basipterygoid processes to basioccipital (0); narrow, length greater than width (1).

32) Posterior-most location of parasphenoid dentition: present anterior to transverse flange of pterygoid exclusively (0); posterior to transverse flange of pterygoid (1); teeth absent on body (2).

33) **Squamosal shape: wide and tapering dorsally, occupies large area of temporal region (0); wide, anterior edge concave (1); narrow, posttemporal process narrowed ventrally (2). Modified from Maddin et al.2008**

34) Position of basicranial joint: in line with or posterior to transverse flange of pterygoid (0); anterior to transverse flange of pterygoid (1).

35) Shape of basipterygoid process: wing-like, articulating surface along anterior edge (0); knob-like, articulating surface located anterolaterally (1).

36) Distribution of palatal dentition: two anterior fields separate (0); two anterior fields confluent (1).

37) Shape of transverse flange of pterygoid: directed laterally or posterolaterally (0); directed anterolaterally (1).

38) **Anterolateral dental field on pterygoid: extending far posteromedially towards basicranial area (0); reduced to a few teeth near suture with palatine(1); absent (2). Modified from Maddin et al 2008.**

OCCIPUT

39) Location of paroccipital process: confined between squamosals, not visible in lateral view (0); posterolateral flange projecting posterior to squamosals, visible in lateral view (1).

40) Paroccipital process of opisthotic-supraoccipital contact: unfused (0); fused (1).

41) Direction of occiput: directed vertically (0); inclined anterodorsally (1).

42) Direction of occipital condyle: directed posteriorly (0); directed sharply ventrally (1).

43) Postparietals: unfused (0); fused (1).

GENERAL SKULL

44) Height of zygomatic arch: inapplicable (0); narrow, less than 30% height of lateral temporal fenestra (1); deep, greater than 50% height of lateral temporal fenestra (0).

45) Lateral temporal fenestra: absent (0); present (1).

46) Longitudinal location of jaw articulation: at level of occipital condyle (0); far posterior to occipital condyle (1).

47) Dorsoventral location of jaw articulation: at level of tooth row (0); below level of tooth row (1).

48) **Skull ornamentation: absent (0); present (1); present with round pits (2). Mofified from Maddin et al 2008.**

49) Lateral temporal fenestra: fenestra absent (0); subcircular (1); subrectangular, posteroventral margin forming acute angle (2); subrectangular (3).

50) Preorbital length of skull: short, less than 1/3 total skull length (0); moderate, between 1/3 and 1/2 skull length (1); long, equal to or greater than 1/2 skull length (2).

51) Head size: large, greater than 30% presacral length (0); small, less than 20% presacral length (1).

52) Frontal length:width ratio: less than 3.3 (0); greater than 3.5 (1).

LOWER JAW

53) Lower jaw shape: lower jaw tapers anteriorly, symphysis thinnest portion of jaw (0); anterior region nearly as

deep as mid-tooth row (1).

54) Snout proportions: wider than tall (0); taller than wide (1).

55) Splenial dorsoventrally deepened at symphysis: absent (0); present (1).

56) Cross-sectional shape of ventral border of angular: ventral margin ridged (0); ventral margin smoothly rounded (1).

57) Splenial contacting posterior coronoid: absent (0); present (1).

58) Position of coronoid eminence of lower jaw: within posterior 1/3 of lower jaw (0); approximately at 40% from anterior of total jaw length (1).

59) Composition of mandibular symphysis: dentary and splenial (0); dentary only (1).

60) Size of Meckelian foramen: small, less than 10% jaw length (0); large, greater than 25% jaw length (1).

61) Number of coronoids in lower jaw: two (0); one (1).

62) Coronoid dentition: absent (0); present (1).

MARGINAL DENTITION

63) Premaxillary tooth number: two (0); three (1); four or greater (2).

64) **Maxillary tooth number: 8–12 teeth (0); 13-21 (1); 22 or more teeth (2). Modified from Maddin et al. 2008**

65) Number of precaniniform maxillary teeth: inapplicable, no canines (0); zero or one precaniniforms (1); three precaniniforms or greater (2).

66) Secondary enlargement in tooth row posterior to caniniforms: absent (0); present (1).

67) Recurvature of marginal teeth: absent (0); present, apex posteriorly recurved (1); highly posteriorly recurved, apex approximately 80–

90 degrees from vertical (2).

68) Relative size of premaxillary tooth #1: premaxillary tooth #1 largest in tooth row (0); premaxillary tooth #1 not largest (1).

69) Caniniform teeth: absent to weakly differentiated (0); present as 2 large maxillary teeth (1); present as enlarged tooth "region", but two pronounced caniniforms absent (2).

70) Number of apical cusps on teeth: single cusp (0); three cuspules (1); greater than three cuspules (2).

71) Bone forming dorsal margin of coronoid region: post-dentary bone (e.g., coronoid, surangular) (0); dentary (1).

72) Shape of lingual surface of marginal dentition: conical, convex (0); spatulate (1).

73) Shoulder on lingual surface: absent (0); present, with lingual curvature (1).

AXIAL SKELETON

74) Shape of dorsal neural arch: not swollen or buttressed (0); swollen or buttressed (1).

75) Number of sacral vertebrae: two or fewer (0); three or greater (1).

76) Sacral rib articulation: 2nd rib primarily braces first, contact with ilium minimal (0); all ribs attach directly to ilium (1).

77) First sacral rib shape: forms flat plate distally (0); U-shaped distally, extremely broad (1).

78) Sacral rib, mode of attachment to vertebrae: sutural in adult (0); fused in adult (1).

79) Expanded rib cage: absent, dorsal ribs restricted ventral to transverse processes (0); present, dorsal ribs rising above transverse processes (1).

80) Size of axis neural spine: large, maximum anteroposterior length greater than that of axis centrum length (0); small, maximum anteroposterior length less than that of axis centrum length (1).

PECTORAL GIRDLE

81) Angle of head of interclavicle: low angle, shallow (0); steep angle, sharply upturned anteriorly (1).

82) Shape of ventral plate of clavicle: narrow, only slightly wider than dorsal process (0); broad, much wider than dorsal process (1).

83) Posterior coracoid triceps process: absent (0); present (1).

84) Supraglenoid foramen of scapula: absent (0); present (1).

85) Number of scapulocorocoid ossifications: two (0); three (1).

86) Notch in anterior edge of scapula: absent (0); present (1).

FORELIMB

87) Ectepicondylar foramen of humerus: absent (0); present (1).

88) Distinct shaft on humerus: absent (0); present (1).

89) Number of manual phalanges in digit II: three (0); two (1).

90) Number of manual phalanges in digit III: four (0); three (1).

91) Number of manual phalanges in digit IV: five (0); four or fewer (1).

92) Number of manual phalanges in digit V: three (0); two (1).

93) **Ratio of length of 4th metacarpal to radius: less than 25% (0); between 30% and 49% (1); equal or greater than 50% (2). Modified from Maddin et al 2008.**

94) Metapodial shape: long and slender, two to three times longer than maximal width (0); short and fat, with small diaphysis (1).

95) Shape of ungual phalanx: strongly recurved (0); weakly recurved (1).

PELVIC GIRDLE

96) Plate-like dorsal process of ilium: absent, height above center of acetabulum less than 50% length to distal tip of posterior process (0); present, height above acetabulum greater than 75% length to posterior process (1).

97) Length of posterior process of ilium: confined above acetabulum (0); projecting posterior to acetabulum (1).

98) External shelf on lateral surface of ilium: absent (0); present (1).

99) Puboischiatic symphysis: evenly distributed between pubis and ischium, lacking enlarged region on pubis (0); enlarged symphyseal region on pubis (1).

100) Lateral pubic tubercle: absent (0); present (1).

101) Pectineal ridge of pubis: absent (0); present (1).

HIND LIMB

102) Shape of posterior ridge of femur: absent, internal fossa without posterior border (0); present, Y-shaped trochanter formed proximally (1).

103) Number of pedal phalanges in digit II: three (0); two (1).

104) Number of pedal phalanges in digit III: four (0); three (1); two (2).

105) Number of pedal phalanges in digit IV: five (0); two (1).

106) Number of pedal phalanges in digit V: four (0); three (1); two (2).

B) CHARACTER CODING FOR *EOCASEA.* See Maddin et al., 2008 [7] for character coding for other taxa

??????0??0 00100??0?1 ?0????02?? ??2???0??0 ??0?1??21? ?????0???? ?????????0

1000011?01 ?????????? ????1110?? 000000
