# Supplementary material for: The Oldest Caseid Synapsid from the Late Pennsylvanian of Kansas, and the Evolution of Herbivory in Terrestrial Vertebrates
Source: PLoS One. 2014 Apr 16;9(4):e94518. doi: 10.1371/journal.pone.0094518 (PMC3989228; doi:10.1371/journal.pone.0094518)
Supplement: Appendix S3 — Caseid Phylogenetic Analysis: PAUP results. (PDF) [file pone.0094518.s003.pdf]

```
Branch-and-bound search completed:
   Score of best tree found = 196
   Number of trees retained = 1
   Time used = 0.02 sec

Tree number 1 (rooted using user-specified outgroup)

/------------------------------------------------------------- Reptilia
|
|                                                   /--------- Diadectes
+--------------------------------------------------+
|                                                   \--------- Limnoscelis
|
|                                                              /--------- Mycterosaurus
|        /-----------------------------------------------------+
|        |                                                     \--------- Varanops
|        |
|        |        /------------------------------------------------------ Eothyris
\--------+        |
         |        |                 /------------------------------------ Oromycter doleso
         |        |                 |
         |        |                 |        /--------------------------- Casea broilii
         \--------+        /--------+        |
                  |        |        |        |                 /--------- Cotylorhynchus
                  |        |        \--------+        /--------+
                  |        |                 |        |        \--------- Angelosaurus
                  \--------+                 \--------+
                           |                          \------------------ Ennatosaurus tec
                           |
                           \--------------------------------------------- Eocasea

Tree description:

  Unrooted tree(s) rooted using outgroup method
  Optimality criterion = parsimony
    Character-status summary:
     106 included characters:
        All characters are of type 'unord'
        All characters have equal weight
        8 characters are parsimony-uninformative
        Number of (included) parsimony-informative characters = 98
    Gaps are treated as "missing"
    Multistate taxa interpreted as polymorphism ("min" values for CI, RI, and RC are
      minimum-possible character lengths)

    Character-state optimization: Accelerated transformation (ACCTRAN)

Tree number 1 (rooted using user-specified outgroup)

Branch lengths and linkages for tree #1 (unrooted)
```

Node	Connected to node	Assigned branch length	Minimum possible length	Maximum possible length
Reptilia (1)*	25	7	2	12
16	25	14	9	19
Diadectes (2)*	16	11	9	14
Limnoscelis (3)*	16	9	7	11
24	25	22	11	25
17	24	17	8	21
Mycterosaurus (4)	17	4	4	7
Varanops (5)	17	10	7	13
23	24	14	4	25
Eothyris (6)	23	4	4	10
22	23	18	5	32

```
    21                  22                 11             2           35
Oromycter doleso (7)    21                  0             0            0
    20                  21                  4             1           33
Casea broilii (8)       20                  3             2            9
    19                  20                 18            14           29
    18                  19                  7             3           12
Cotylorhynchus (10)     18                  2             0            8
Angelosaurus (11)       18                  1             1            3
Ennatosaurus tec (12)   19                  4             2            9
Eocasea    (13)         22                  2             2            3
-------------------------------------------------------------------------
Sum                                       182
```

Data matrix and reconstructed states for internal nodes:

```
                           111111111122222222223333333333444444444455555555556666666666777
Taxon/Node        123456789012345678901234567890123456789012345678901234567890123456789012
------------------------------------------------------------------------------------------
Reptilia          010000000000000000100001000200012000000000000000000000000001000102100010000
                                    1      1 1           1       1             1  2   1
Diadectes         000000000000000000100010112000002000012010110001100000000010110200001 0?01
                                    1
Limnoscelis       100000000000000000110000011000000?000002010110000000201101010010010000000000
Mycterosaurus     01010001112010110111121110211110110000?011001100110010100000000021202110 00
                                                        111 1111        1
Varanops          010000021120101000102111122121011000000011001110020010101101000212021100 0
                                                     1      1                           1
Eothyris          100100011100100001001001010010 0?100000111002100110010000010000101111 1000
                       1                       1      1    1        1     1
Oromycter doleso  100?000111?000001000?0000????00??0000000000?0002??00100000000010000 00001
                   1  11      111  111 1111    11  1111 11111 111   11 111111111        1
Casea broilii     1002001111101000000010010 00?1100?101101111111012111010000100010000000111
                    1  1          1 1   1           1                     1
Cotylorhynchus    1012111221111110110012101000221 0?2110011111011012311010101100101000000101
Angelosaurus      000?000??0?000000000?0000????100?1100101110?000???000000000000?0?0000201
                  111 111  1 111111111 1111             1   1 111   11111111 111     11 1
Ennatosaurus tec  101211122111111011011211100022 01?20011111100110123110101001000000000000211
                                                 1   11      1                  1
Eocasea           000?000??00010000001?0000?02?00?20000?00000?10021?000000000000?0?0?00010
                  111 111        11 1   111    11  111  1 11   11   11111 111111 1 1 11
16                000000000000000000110000011000002000002010110000100000000100100200001 0000
17                010100011120101001102111021111011000000011001100110010101101000212021100 0
18                1012111221111110110012101000221002110011111011012311010101100101000000201
19                1012111221111110110012101000221002111111111011012311010101100100000000211
20                101201111110100010011001000211002111101111011012111010001100000000000111
21                100201011110100010011001000211002111101111011012111010001100001000000011
22                100201011100100010011001000211002111001111011012111010001100001000000010
23                100101011100100001001001010011001000001111011001101100001100001000011000
24                000100011100100001101001010011001000000110011001100100001000002000011000
25                000000000000000000110000001000002000000010000000100000000100000200001 0000
```

Data matrix and reconstructed states for internal nodes (continued):

```
                                             1111111
                  7777777888888888899999999990000000
Taxon/Node        3456789012345678901234567890123456
----------------------------------------------------
Reptilia          0000100000011001000010001000110000
                   1   1 1 11               11
Diadectes         01000110000100000000?1111111010000
                           1
Limnoscelis       0101010001010000000001111110010000
                      1   1
Mycterosaurus     00001000000000010000?0001001010?0?
```

```
                     1 11 111 1   1111     11 1 1 1
Varanops          0001110001011001000020101000110000
                                  111
Eothyris          00000000000000000000?0000000000?0?
                   1111111111111111111 1111111111 1
Oromycter doleso  10000000000000000000?0100000000?0?
                   1111111111111111111 1 11111111 1
Casea broilii     1011011111101001000010011000100?01
                                  1111  1       1 1
Cotylorhynchus    1011011110101111111101110000101111
Angelosaurus      1011011010101111000001110000101212
                         1        1111
Ennatosaurus tec  1011011001101001110101010000101112
                         11         1   1
Eocasea           00011001000000000000?0111000000000
                       1  111111111111 1   1  11
16                0100010001010000000011111110010000
17                0001110001001001111020101000110000
18                1011011110101111111101110000101112
19                1011011111101001111101110000101112
20                1011011111101001111110111000101111
21                1011011111101001111110111000101111
22                0001110111101001111110111000100000
23                0001110111101001111110111000100000
24                0001110001001001111010101000110000
25                0000110001011001000010101000110000


Tree length = 196
Consistency index (CI) = 0.7296
Homoplasy index (HI) = 0.3367
CI excluding uninformative characters = 0.7151
HI excluding uninformative characters = 0.2849
Retention index (RI) = 0.7452
Rescaled consistency index (RC) = 0.5437

/------------------------------------------------------------------------ Reptilia
|
|                                                              /--------- Diadectes
+-------------------------------------------------------------16
|                                                              \--------- Limnoscelis
|
25                                                             /--------- Mycterosaurus
|        /----------------------------------------------------17
|        |                                                     \--------- Varanops
|        |
|        |        /------------------------------------------------------ Eothyris
\-------24        |
         |        |                 /------------------------------------ Oromycter doleso
         |        |                 |
         |        |                 |        /--------------------------- Casea broilii
         \-------23        /-------21        |
                  |        |        |        |                 /--------- Cotylorhynchus
                  |        |        \-------20        /-------18
                  |        |                 |        |        \--------- Angelosaurus
                  \-------22                 \-------19
                           |                          \------------------ Ennatosaurus tec
                           |
                           \--------------------------------------------- Eocasea


Apomorphy lists:

               Branch                 Character   Steps     CI   Change
-----------------------------------------------------------------------
         node_25 --> Reptilia         2 (2.)          1  0.500  0 --> 1
                                      23 (24.)        1  0.333  0 --> 1
                                      27 (28.)        1  0.667  0 --> 2
```

	31 (32.)	1	0.500	0 ==> 1
	61 (62.)	1	0.333	0 --> 1
	64 (65.)	1	0.500	0 --> 1
	95 (96.)	1	0.500	1 ==> 0
node_25 --> node_16	25 (26.)	1	0.500	0 ==> 1
	38 (39.)	1	1.000	0 ==> 2
	42 (43.)	1	0.500	0 --> 1
	43 (44.)	1	0.500	0 ==> 1
	60 (61.)	1	1.000	0 ==> 1
	74 (75.)	1	1.000	0 --> 1
	77 (78.)	1	0.500	1 --> 0
	85 (86.)	1	1.000	1 ==> 0
	88 (89.)	1	1.000	1 ==> 0
	94 (95.)	1	0.500	0 ==> 1
	96 (97.)	1	0.500	0 --> 1
	98 (99.)	1	1.000	0 ==> 1
	99 (100.)	1	1.000	0 --> 1
	101 (102.)	1	1.000	1 ==> 0
node_16 --> Diadectes	22 (23.)	1	0.333	0 ==> 1
	24 (25.)	1	0.500	0 --> 1
	26 (27.)	1	0.750	1 ==> 2
	37 (38.)	1	0.333	0 ==> 1
	47 (48.)	1	0.500	0 ==> 1
	57 (58.)	1	0.500	1 ==> 0
	58 (59.)	1	0.500	0 ==> 1
	61 (62.)	1	0.333	0 --> 1
	72 (73.)	1	0.500	0 ==> 1
	79 (80.)	1	0.500	0 ==> 1
	100 (101.)	1	0.667	0 ==> 1
node_16 --> Limnoscelis	1 (1.)	1	0.500	0 ==> 1
	48 (49.)	1	0.750	1 ==> 0
	50 (51.)	1	1.000	0 ==> 2
	52 (53.)	1	0.333	0 --> 1
	53 (54.)	1	0.500	0 ==> 1
	55 (56.)	1	0.500	0 ==> 1
	63 (64.)	1	0.500	2 ==> 1
	68 (69.)	1	0.500	1 ==> 0
	76 (77.)	1	0.500	0 --> 1
node_25 --> node_24	4 (5.)	1	0.667	0 --> 1
	8 (9.)	1	0.667	0 ==> 1
	9 (10.)	1	1.000	0 ==> 1
	10 (11.)	1	0.500	0 ==> 1
	13 (14.)	1	1.000	0 ==> 1
	21 (22.)	1	0.667	0 --> 1
	24 (25.)	1	0.500	0 --> 1
	29 (30.)	1	0.667	0 ==> 1
	30 (31.)	1	1.000	0 ==> 1
	32 (33.)	1	1.000	2 --> 0
	33 (34.)	1	1.000	0 ==> 1
	41 (42.)	1	1.000	0 ==> 1
	44 (45.)	1	1.000	0 ==> 1
	45 (46.)	1	1.000	0 ==> 1
	49 (50.)	1	1.000	0 ==> 1
	52 (53.)	1	0.333	0 --> 1
	69 (70.)	1	0.667	0 --> 1
	76 (77.)	1	0.500	0 --> 1
	84 (85.)	1	0.500	1 --> 0
	89 (90.)	1	1.000	0 --> 1
	90 (91.)	1	1.000	0 --> 1
	91 (92.)	1	1.000	0 --> 1
node_24 --> node_17	2 (2.)	1	0.500	0 --> 1
	11 (12.)	1	1.000	0 ==> 2
	15 (16.)	1	1.000	0 ==> 1
	21 (22.)	1	0.667	1 --> 2
	22 (23.)	1	0.333	0 ==> 1
	23 (24.)	1	0.333	0 --> 1
	26 (27.)	1	0.750	1 ==> 2
	27 (28.)	1	0.667	0 --> 1

	28 (29.)	1	1.000	0 ==> 1
	32 (33.)	1	1.000	0 --> 1
	54 (55.)	1	1.000	0 ==> 1
	56 (57.)	1	1.000	0 --> 1
	59 (60.)	1	1.000	0 --> 1
	64 (65.)	1	0.500	0 --> 1
	65 (66.)	1	1.000	0 ==> 2
	67 (68.)	1	1.000	0 ==> 2
	93 (94.)	1	1.000	1 --> 2
node_17 --> Mycterosaurus	16 (17.)	1	0.500	0 ==> 1
	20 (21.)	1	0.333	0 ==> 1
	95 (96.)	1	0.500	1 ==> 0
	100 (101.)	1	0.667	0 ==> 1
node_17 --> Varanops	4 (5.)	1	0.667	1 --> 0
	8 (9.)	1	0.667	1 ==> 2
	18 (19.)	1	0.500	1 ==> 0
	25 (26.)	1	0.500	0 ==> 1
	27 (28.)	1	0.667	1 --> 2
	29 (30.)	1	0.667	1 ==> 2
	46 (47.)	1	1.000	0 ==> 1
	48 (49.)	1	0.750	1 ==> 0
	49 (50.)	1	1.000	1 ==> 2
	84 (85.)	1	0.500	0 --> 1
node_24 --> node_23	1 (1.)	1	0.500	0 ==> 1
	6 (7.)	1	1.000	0 --> 1
	19 (20.)	1	0.500	1 --> 0
	39 (40.)	1	1.000	0 ==> 1
	42 (43.)	1	0.500	0 --> 1
	51 (52.)	1	1.000	0 --> 1
	58 (59.)	1	0.500	0 ==> 1
	63 (64.)	1	0.500	2 ==> 1
	80 (81.)	1	1.000	0 --> 1
	81 (82.)	1	1.000	0 --> 1
	83 (84.)	1	1.000	0 --> 1
	92 (93.)	1	1.000	0 --> 1
	96 (97.)	1	0.500	0 --> 1
	102 (103.)	1	1.000	1 --> 0
node_23 --> Eothyris	44 (45.)	1	1.000	1 ==> 2
	65 (66.)	1	1.000	0 ==> 1
	66 (67.)	1	1.000	0 ==> 1
	67 (68.)	1	1.000	0 ==> 1
node_23 --> node_22	4 (5.)	1	0.667	1 --> 2
	17 (18.)	1	1.000	0 --> 1
	18 (19.)	1	0.500	1 ==> 0
	20 (21.)	1	0.333	0 --> 1
	26 (27.)	1	0.750	1 --> 0
	28 (29.)	1	1.000	0 ==> 2
	33 (34.)	1	1.000	1 ==> 2
	34 (35.)	1	1.000	0 --> 1
	35 (36.)	1	1.000	0 --> 1
	36 (37.)	1	0.500	0 --> 1
	47 (48.)	1	0.500	0 --> 1
	48 (49.)	1	0.750	1 ==> 2
	50 (51.)	1	1.000	0 --> 1
	52 (53.)	1	0.333	1 --> 0
	53 (54.)	1	0.500	0 --> 1
	68 (69.)	1	0.500	1 --> 0
	69 (70.)	1	0.667	1 --> 0
	71 (72.)	1	0.500	0 ==> 1
node_22 --> node_21	11 (12.)	1	1.000	0 --> 1
	37 (38.)	1	0.333	0 --> 1
	72 (73.)	1	0.500	0 ==> 1
	73 (74.)	1	1.000	0 ==> 1
	75 (76.)	1	1.000	0 --> 1
	77 (78.)	1	0.500	1 --> 0
	79 (80.)	1	0.500	0 --> 1
	103 (104.)	1	1.000	0 --> 1
	104 (105.)	1	1.000	0 --> 1

	105 (106.)	1	1.000	0 --> 1
	106 (107.)	1	0.667	0 --> 1
node_21 --> node_20	3 (4.)	1	1.000	0 --> 1
	7 (8.)	1	1.000	0 ==> 1
	63 (64.)	1	0.500	1 --> 0
	70 (71.)	1	0.667	0 --> 1
node_20 --> Casea broilii	20 (21.)	1	0.333	1 --> 0
	43 (44.)	1	0.500	0 ==> 1
	62 (63.)	1	1.000	0 ==> 1
node_20 --> node_19	5 (6.)	1	1.000	0 ==> 1
	8 (9.)	1	0.667	1 ==> 2
	9 (10.)	1	1.000	1 ==> 2
	12 (13.)	1	1.000	0 ==> 1
	14 (15.)	1	1.000	0 ==> 1
	16 (17.)	1	0.500	0 ==> 1
	21 (22.)	1	0.667	1 --> 2
	22 (23.)	1	0.333	0 ==> 1
	29 (30.)	1	0.667	1 ==> 2
	38 (39.)	1	1.000	0 ==> 1
	49 (50.)	1	1.000	1 ==> 3
	55 (56.)	1	0.500	0 ==> 1
	61 (62.)	1	0.333	0 --> 1
	70 (71.)	1	0.667	1 --> 2
	93 (94.)	1	1.000	1 ==> 0
	94 (95.)	1	0.500	0 ==> 1
	97 (98.)	1	1.000	1 ==> 0
	106 (107.)	1	0.667	1 --> 2
node_19 --> node_18	36 (37.)	1	0.500	1 --> 0
	37 (38.)	1	0.333	1 --> 0
	63 (64.)	1	0.500	0 --> 1
	71 (72.)	1	0.500	1 --> 0
	82 (83.)	1	1.000	1 ==> 0
	86 (87.)	1	1.000	0 ==> 1
	87 (88.)	1	1.000	0 ==> 1
node_18 --> Cotylorhynchus	70 (71.)	1	0.667	2 --> 1
	106 (107.)	1	0.667	2 --> 1
node_18 --> Angelosaurus	104 (105.)	1	1.000	1 ==> 2
node_19 --> Ennatosaurus tec	19 (20.)	1	0.500	0 --> 1
	23 (24.)	1	0.333	0 --> 1
	31 (32.)	1	0.500	0 ==> 1
	57 (58.)	1	0.500	1 ==> 0
node_22 --> Eocasea	10 (11.)	1	0.500	1 ==> 0
	40 (41.)	1	1.000	1 ==> 0
