# Supplementary material for: The Oldest Caseid Synapsid from the Late Pennsylvanian of Kansas, and the Evolution of Herbivory in Terrestrial Vertebrates
Source: PLoS One. 2014 Apr 16;9(4):e94518. doi: 10.1371/journal.pone.0094518 (PMC3989228; doi:10.1371/journal.pone.0094518)
Supplement: Appendix S5 — Character coding and PAUP results based on Benson 2012+ Eocasea. (PDF) [file pone.0094518.s005.pdf]

Character coding for Eocasea, as used in analysis of Benson 2013.
Eocasea ?????110?0 10????????? ?????0????? ????????000 ??????????? ?????????00
0?100??000 000000001?2 01010000?12 ??00?????? ??????????? ??????0????
000????0??? ??0?0??1??2 00000?00000 00000000000
0????????? ?????????? ????????? ?????0???? ??1{01}2?01? 0?000??0?? 000?1??0

PAUP results for synapsid phylogeny using data from Benson 2012, with character coding listed above included.

P A U P *
Version 4.0b10 for Macintosh (PPC/Altivec)
Data matrix has 54 taxa, 239 characters
Valid character-state symbols: 01234
Missing data identified by '?'
Gaps identified by '-'

Processing of file "Benson 2012 pelycosaurs+Eoc.dat" completed.

Outgroup status changed:
  4 taxa transferred to outgroup
  Total number of taxa now in outgroup = 4
  Number of ingroup taxa = 50

Heuristic search settings:
  Optimality criterion = parsimony
    Character-status summary:
      Of 239 total characters:
        All characters are of type 'unord'
        All characters have equal weight
        1 character is parsimony-uninformative
        Number of parsimony-informative characters = 238
    Gaps are treated as "missing"
    Multistate taxa interpreted as polymorphism
  Starting tree(s) obtained via stepwise addition
  Addition sequence: simple (reference taxon = Tseajaia campi)
  Number of trees held at each step during stepwise addition = 1
  Branch-swapping algorithm: tree-bisection-reconnection (TBR)
  Steepest descent option not in effect
  Initial 'MaxTrees' setting = 50100 (will be auto-increased by 100)
  Branches collapsed (creating polytomies) if maximum branch length is zero
  'MulTrees' option in effect
  Topological constraints not enforced
  Trees are unrooted

Heuristic search completed
  Total number of rearrangements tried = 555170346
  Score of best tree(s) found = 726

Number of trees retained = 11664
Time used = 00:05:38.0

Strict consensus of 11664 trees:

```
                                                   /------ Tseajaia campi
/--------------------------------------------------+
|                                                  \------ Limnoscelis
|
|                                                 /------ Captorhinus
+-------------------------------------------------+
|                                                 \------ Protorothyris archeri
|
|                                                  /------ Dimetrodon spp.
|                                                  |
|                                                  +------ Secodontosaurus
|                                                  |
|                                                  +------ Ctenorhachis
|                                    /-------------+
|                                    |             +------ Sphenacodon ferox
|                                    |             |
|                                    |             +----- Cryptovenator
|                                    |             |
|                                    |             \------ Cutleria
|                                /---+
|                                |   |         /------ Titanophoneus
|                                |   |      /--+
|                                |   |      |  \------ Biseridens
|                                |   |   /--+
|                           /---+   |   |  \------------ Biarmosuchus tener
|                           |   |   \---+
|                           |   |       \---------------- Raranimus
|                       /---+   |
|                       |   |   \------------------------- Pantelosaurus
|                       |   |
|                       |   \------------------------------ Haptodus garnettensis
|                       |
|                       |                       /------ Edaphosaurus boanerges
|                   /---+                     /—+
|                   |   |                     | \------ Edaphosaurus novomexicanus
|                   |   |                     |
|                   |   |                 /---+------------ Lupeosaurus kayi
|                   |   |                 |   |
|              /----+   \-----------------+   \------------ Glaucosaurus
```

```
|           |   |                   |
|           |   |                   \----------------- Ianthasaurus
|           |   |
|       /---+   \----------------------------------------- Ianthodon
|       |   |
|       |   |                       /----------------- Archaeothyris
|       |   |                       |
|       |   \-----------------------+   /------------ Varanosaurus acutirostris
|       |                           |   |
|       |                           \---+   /------ Ophiacodon spp.
|       |                               \--+
|       |                                   \------ Stereophallodon
|       |
|       |                                   /------ Watongia
|       |                               /---+
|       |                               |   \------ Varanodon
|       |                           /---+
|       |                           |   \----------- Varanops
|   /---+                           |
|   |   |                       /---+       /------ Ruthiromia
|   |   |                       |   |       |
|   |   |                       |   \-------+------ Aerosaurus wellesi
|   |   |                       |           |
|   |   |                       |           \------ Aerosaurus greenleorum
|   |   |                       |
|   |   |                       +-------------------- Mesenosaurus
|   |   |                       |
|   |   |                   /---+-------------------- Basicranodon
|   |   |                   |   |
|   |   |                   |   +-------------------- Mycterosaurus
|   |   |                   |   |
|   |   |                   |   +-------------------- Heleosaurus
|   |   \-------------------+   |
|   |                       |   +-------------------- BP 1 5678 Elliot
|   |                       |   |
|   |                       |   \--------------------- Pyozia
|   |                       |
|   |                       \------------------------- Archaeovenator
|   |
\---+                               /---------------- Echinerpeton
    |                               |
    |                               |   /----------- Oromycter
    |                               |   |
    |                           /---+   |   /------ Ennatosaurus
    |                           |   |   |   |
    |                           |   |   |   +------ Casea broilii
    |                           |   |   |   |
    |                           |   \---+   +------ Cotylorhynchus romeri
    |                           |       |   |
```

```
|                          |        |    +------ Angelosaurus romeri
|                          |        |    |
|                          |        |    +------ Angelosaurus dolani
|                          |        \----+
|                      /—+              +------ Caseopsis agilis
|                      |   |              |
|                      |   |              +------ Trichasaurus
|                      |   |              |
|                      |   |              +------ Cotylorhynchus halselli
|                      |   |              |
|                      |   |              +------ Cotylorhynchus bransoni
\— — — — —  ------------+   |              |
                       |   |              \------ Casea rutena
                       |   |
                       |   \-------------------  Eocasea
                       |
                       |                  /------ Eothyris
                       \-----------------+
                                          \------ Oedaleops
```

Tree description:

Unrooted tree(s) rooted using outgroup method
Optimality criterion = parsimony
Character-status summary:
Of 239 total characters:
All characters are of type 'unord'
All characters have equal weight
1 character is parsimony-uninformative
Number of parsimony-informative characters = 238
Gaps are treated as "missing"
Multistate taxa interpreted as polymorphism ("min" values for CI, RI, and RC are minimum-possible character lengths)

Character-state optimization: Accelerated transformation (ACCTRAN)

Tree number 1 (rooted using user-specified outgroup)

Tree length = 726
Consistency index (CI) = 0.4545
Homoplasy index (HI) = 0.5579
CI excluding uninformative characters = 0.4538
HI excluding uninformative characters = 0.5462
Retention index (RI) = 0.7516
Rescaled consistency index (RC) = 0.3416
