# Supplementary material for: The Oldest Caseid Synapsid from the Late Pennsylvanian of Kansas, and the Evolution of Herbivory in Terrestrial Vertebrates
Source: PLoS One. 2014 Apr 16;9(4):e94518. doi: 10.1371/journal.pone.0094518 (PMC3989228; doi:10.1371/journal.pone.0094518)
Supplement: Appendix S6 — Eocasea specimen measurements. (PDF) [file pone.0094518.s006.pdf]

Eocasea specimen measurements

Pineal diameter = 1.9.mm
Parietal width = 6.35 mm
Vertebral centrum length
- Anterior dorsal= 2.35 mm
- Mid-dorsal= 2.9 mm
- Posterior dorsal= 2.59 mm
- Anterior caudal= 2.1 mm
- Mid-caudal = 2.6mm

Femur Length = 15.02 mm
- Mid-shaft diameter= 2.25 mm

Tibia Length = 10.7 mm

Ilium Height = 9.1 mm
Ilium neck = 2.7 mm
Ischium Length = 8.1 mm
Pubis Length = 4.25mm
